# Cytotoxic effects of *Euterpe oleracea* Mart. in malignant cell lines

**DOI:** 10.1186/1472-6882-14-175

**Published:** 2014-05-29

**Authors:** Dulcelena Ferreira Silva, Flávia Castello Branco Vidal, Debora Santos, Maria Célia Pires Costa, José Andrés Morgado-Díaz, Maria do Desterro Soares Brandão Nascimento, Roberto Soares de Moura

**Affiliations:** 1 Morphology Department, Federal University of Maranhão, Rua Coelho Neto nº 311, Centro, São Luís, Maranhão 65020-140, Brazil; 2 Tumors and DNA Bank from Maranhão, Federal University of Maranhão, Maranhão, Brazil; 3 Biology and Chemistry Department, State University of Maranhão, Maranhão, Brazil; 4 Structural Biology Laboratory, Cell Biology Division, National Cancer Institute José Alencar Gomes da Silva, Rio de Janeiro, Brazil; 5 Pathology Department, Federal University of Maranhão, Maranhão, Brazil; 6 Laboratory of Pharmacology and Psychobiology, Pharmacology Department, State University of Rio de Janeiro, Rio de Janeiro, Brazil

**Keywords:** Anticancer, *Euterpe oleracea* mart., MCF-7, Phytochemicals, Chemopreventive

## Abstract

**Background:**

*Euterpe oleracea* Mart., a plant from the Amazon region, is commonly known as açaí or juçara; it has high nutritional value and elevated levels of lipids, proteins, and minerals. Açaí is an abundant and much consumed fruit by the Amazon local population, and studies have demonstrated that it is rich in phytochemicals with antioxidant, anti-inflammatory, and anticancer activities. Therefore, the aim of this study was to test this plant for anticancer activity in different human malignant cell lines.

**Methods:**

Cell lines derived from breast and colorectal adenocarcinomas were treated with 10, 20, and 40 μg/mL of bark, seed, and total açaí fruit hydroalcoholic extracts for 24 and 48 h. After treatment, cell viability was measured using 3-(4,5-dimethylthiazol-2-yl)-2,5-diphenyltetrazolium bromide (MTT) assays, and cell morphological features were observed by light and transmission electron microscopy. The type of cell death was also evaluated. The data were analyzed statistically by one-way analysis of variance (ANOVA), followed by Dunnett’s or Tukey’s *post hoc* tests, as appropriate.

**Results:**

We observed that of all the cell lines tested, MCF-7 was the only line that responded to açaí treatment. The extracts caused significant reduction (p < 0.01) in cell viability and altered cell morphological features by inducing the appearance of autophagic vacuoles, as observed by transmission electron microscopy. Furthermore, increased expression of LC3BII, a protein marker of autophagosome formation, was observed by western blotting. Caspase Glo™ assays and morphologic observations by DAPI nuclear staining and transmission electron microscopy did not indicate any apoptotic events.

**Conclusions:**

The present study demonstrated that açaí possesses antitumorigenic potential in the MCF-7 cell line. Further studies are needed to identify the compound (s) responsible for this cytotoxic activity and the molecular target in the cell. This discovery of the anticancer potential of açaí may help in the development of chemopreventive drugs and may have therapeutic effects in the treatment of breast cancer.

## Background

*Euterpe oleracea* Mart. is an indigenous monocot plant found in the estuary of the Amazon region and is commonly known as juçara or acai. It is widely consumed by the Amazonian population living on the shore of the Amazon River [1].

Besides its high macronutrient content, açaí has been shown to possess high levels of phytochemicals with antioxidant, anti-inflammatory, hypocholesterolemic, and anticancer activities [2-6]. Açaí was found to inhibit the production of reactive oxygen species and the activity of cyclooxygenases 1 and 2 [7]. In rats, açaí extracts induces endothelium-dependent vasodilation [8]. An *in vivo* study with healthy volunteers demonstrated that açaí pulp caused a significant increase in the antioxidant capacity of plasma, which indicates the *in vivo* antioxidant potential of *E. oleracea* Mart. [9].

Additionally, a few studies have demonstrated the antitumorigenic activity of açaí. One study showed that açaí’s polyphenolic, glycoside, and aglycone forms could induce apoptosis in HL-60 leukemia cells [10]. An *in vivo* study reported that açaí intake could attenuate dimethylhydrazine-induced colon carcinogenesis in rats [11].

Due to the wide usage of açaí by a local population of the Amazon region and the antitumorigenic potential of this plant, we investigated whether açaí extracts from the fruit, bark, and seed possess anticancer activity in human malignant cell lines. MTT viability assays as well as morphologic analysis of cells performed using light and transmission electron microscopy were performed, and the type of cell death was also analyzed.

## Methods

### Plant material and preparation of açaí hydroalcoholic extracts

The specimen was collected at Juçara’s park, an environmental protection area located at São Luís County. The plant material was authenticated by the Rosa Mochel Herbarium from School of Biological Studies, State University of Maranhão, where a voucher specimen was deposited (reference number of 30).

Fruits were harvested and stored at -20°C until use. After defrosting at room temperature, fruits were separated into three portions: bark, seed and total fruit (bark + seed). The procedures used to prepared the hydroalcoholic extracts have been described previously [3]. Briefly, about 360 g of each different portion were washed in running water and boiled in distilled water for 5 minutes. After, they were macerated separately in 400 ml of ethanol PA for 2 hours with intermittent shaking and then kept in dark bottles at 4°C for 10 d. The extracts were filtered thought Whatman no. 1 filter paper, and the ethanol was evaporated under low pressure at 40°C. The extracts were then lyophilized (LIO-TOP model 202; Fisatom Equipamentos, São Paulo, Brazil) and frozen at -20°C until use.

Determination of total polyphenols in açaí extracts was performed by the Folin-Ciocalteau colorimetric method determined on a Spectrophotometer UV/VIS by monitoring the absorbance at 700 nm using gallic acid as a reference standard (50, 100, 150, 250 and 1000 mg/mL). Values were evaluated as the mg equivalent of gallic acid per g of extract [12].

### Cell culture and treatments

The cell lines Caco-2 (ATCC, # HTB-37, Rockville, MD, USA) and HT-29 (ATCC, # HTB-38), both derived from human colon adenocarcinoma, and MCF-7 (ATCC, # HTB-22) and MDA-MB-468 (ATCC, # HTB-132, Rockville, MD, EUA), derived from human mammary adenocarcinoma, were grown in Dulbecco’s modified Eagle’s medium (DMEM) (Invitrogen) supplemented with 10% fetal bovine serum, penicillin G (60 mg/l), and streptomycin (100 mg/l) at 37°C in a humidified atmosphere of 5% CO_2_.

The lyophilized extracts were dissolved in Milli-Q water, and the solution was filtered through a 0.2-μm pore syringe filter and stored at -20°C until use. The cultured cells were treated with 10, 20, and 40 μg/ml of the extracts for 24 and 48 h.

### MTT viability assay

Cells (1 × 10^4^ cell/ml) were cultured in 96-well plates in the presence or absence of the extracts for 24 and 48 h. The supernatant was removed, and 10 μl of 3-(4,5-dimethylthiazol-2-yl)-2,5-diphenyltetrazolium bromide (MTT) in DMEM medium was added to each well. Cells were incubated in a CO_2_ chamber for 3 h with protection from light. The absorbance at 538 nm was measured with a Spectra Max 190 spectrophotometer (Molecular Devices, Sunnyvale, CA, EUA).

### Morphologic analysis

Cells were cultured in 96-well plates in the presence or absence of the extracts for 24 and 48 h. After treatment, the morphological features of the cells were evaluated by phase-contrast microscopy using an inverted microscope Axio Observer Z1, equipped with a camera, AxiocamHRc Ver.3 (Carl Zeiss Inc., Germany).

For transmission electron microscopy analysis, cells were grown in culture flasks treated with açaí extracts for 24 h. Subsequently cells were washed with PBS and fixed for 1 h with Karnovsky fixer (2.5% glutaraldehyde, 4% paraformaldehyde, and 0.1 M cacodylate buffer). Post-fixation was carried out with 1% osmium tetroxide in cacodylate buffer containing 0.8% potassium ferrocyanide and 5 mM CaCl_2_ for 45 min at 4°C. Subsequently, cells were dehydrated in a graded series of acetone and embedded in epoxy resin. Ultrathin sections (60 nm) were stained with uranyl acetate and lead citrate and examined using a Zeiss EM 906 (Jena, Germany) transmission electron microscope.

### Nuclear staining and Caspase-Glo^®^ 3/7 luminescent assay

The nuclear and chromosome counterstain, DAPI (4′,6-diamidino-2-phenylindole), was used for nuclear morphologic observations of treated cells with açaí extracts. To evaluate possible apoptotic activity of the extracts, we used a luminescent kit that measures caspase-3/7 activity (Caspase-Glo™ 3/7, Promega, WI, USA). After treatment with the extracts, 10 μl of Caspase-Glo™ 3/7 reagent was added to the culture, and after 90 min, the luminescence was analyzed in an illuminometer, Veritas Turner Biosystems (Madison, USA).

### Total cell lysate preparation and western blot analysis

Total cell lysates were obtained by incubating cells in lysis buffer (1% Triton X-100, 0.5% sodium deoxycholate, 0.2% SDS, 150 mM NaCl, 2 mM EDTA, 10 mM Hepes [pH 7.4], 20 mM NaF, 1 mM orthovanadate, and a protease-inhibitor cocktail [1:100 dilution]), for 30 min at 4°C. After centrifugation at 10,000 × *g* for 10 min at 4°C, the supernatant was removed and stored at -20°C for subsequent analysis.

Equal amounts of cell protein (40 μg/lane), quantified using the BCA protein assay kit (BioRad, Hercules, CA, USA), were electrophoretically separated by SDS-PAGE on 13% gels and transferred to nitrocellulose membranes. Membranes were blocked and incubated overnight with the primary antibody, anti-LC3B (1:1500) (Sigma Chemical Co., St. Louis, MO, USA), and for 1 h with peroxidase-conjugated goat anti-rabbit (1:10,000). Proteins were visualized using an enhanced chemiluminescence kit (GE Healthcare). α-Tubulin was used as the loading control for each protein.

### Statistical analysis

Statistical analysis was performed using GraphPad Prism 4.0 version for Windows (GraphPad Software, San Diego, CA). The data were analyzed by one-way analysis of variance (ANOVA), followed by Dunnett’s or Tukey’s *post hoc* tests, as appropriate. Results were considered statistically significant for p values < 0.05.

## Results

### Estimation of total polyphenol concentration in açaí hydroalcoholic extracts and effects on cell viability

**Figure 1:**
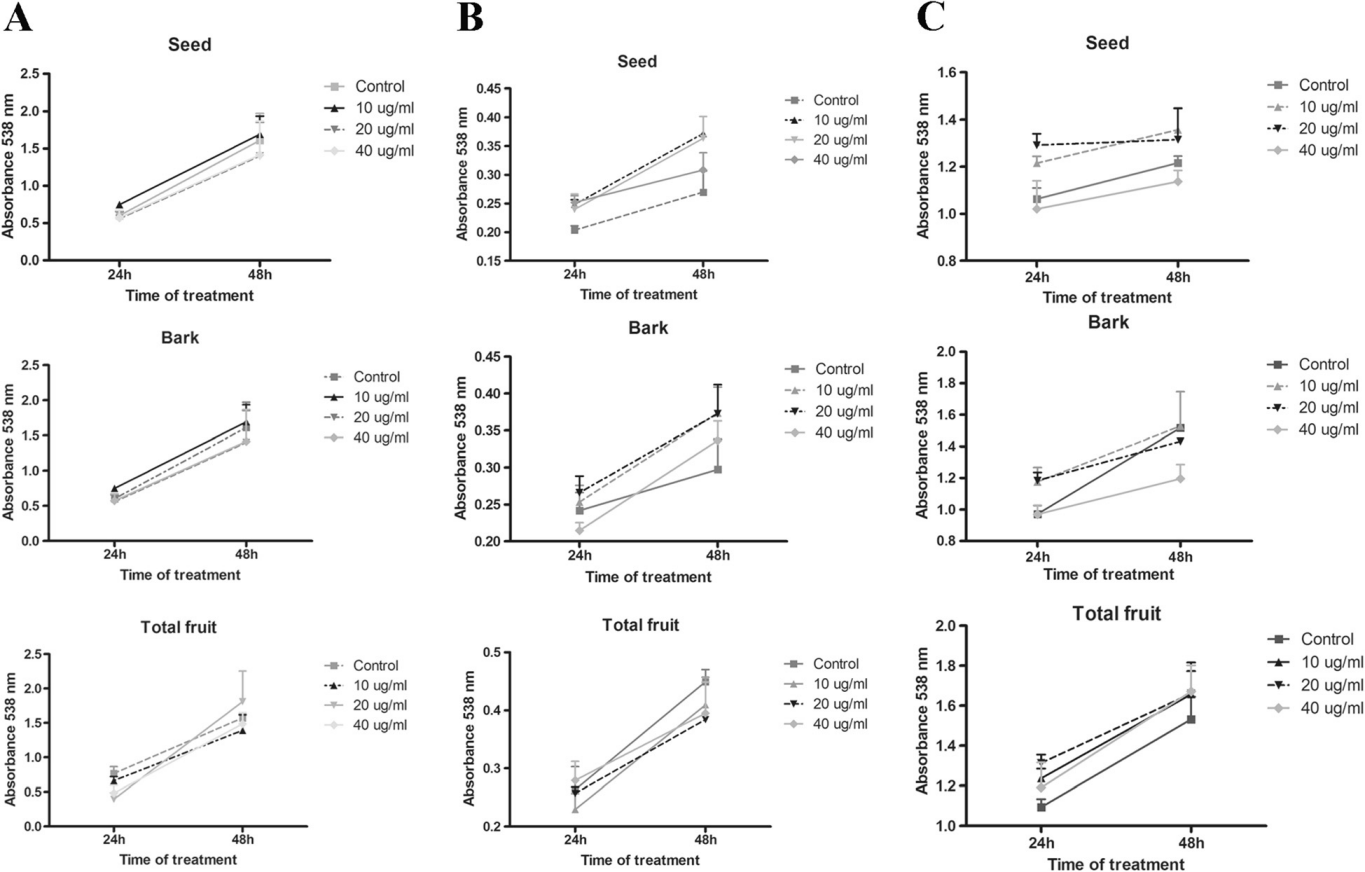
**Were MTT assays performed to analyze the viability of Caco-2 (A), HT-29 (B), and MDA-MB-468 (C) cells after treatment with the bark, seed, or total fruit extracts of açaí.** None of the extracts caused cytotoxicity in these cells lines.

**Figure 2:**
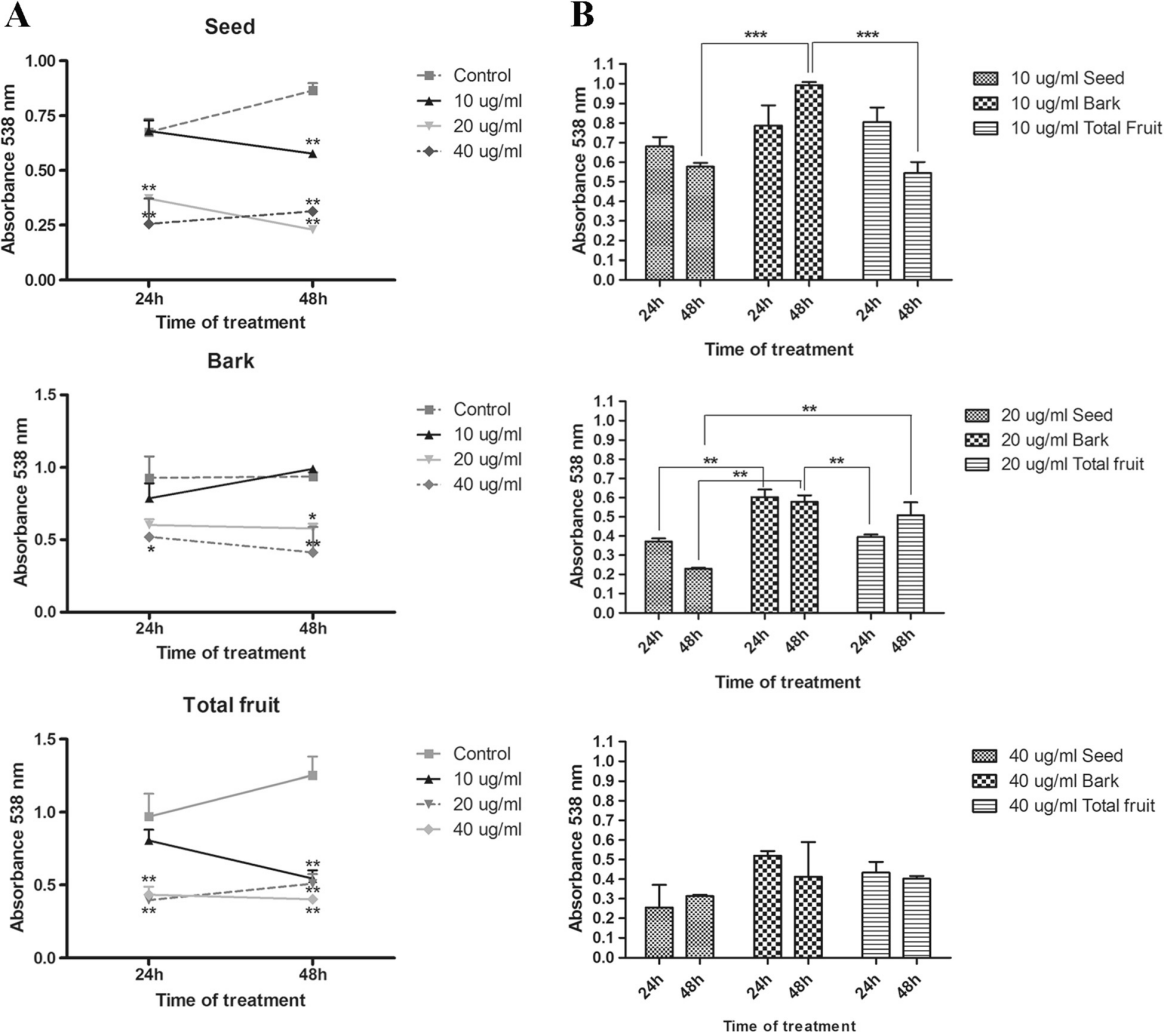
**MTT assay analyzing the viability of MCF-7 cells after treatment with the bark, seed, or total fruit extracts of açaí. (A)** The three hydroalcoholic extracts of açaí caused significant reduction in MCF-7 cell viability after 24 and 48 h of treatment. *p < 0.05, **p < 0.01. One-way analysis of variance (ANOVA) followed by Dunnett’s *post hoc* test. **(B)** Comparison using the same concentration of the three different extracts. Treatment of cells with total fruit and seed extract for 24 and 48 h was significantly more effective than the bark extract in MCF-7 cells. *p < 0.05, **p < 0.01, ***p < 0.001. One-way analysis of variance (ANOVA), followed by Tukey’s *post hoc* test.

Concentration analysis of polyphenols from açaí demonstrated that the seed extract possessed the highest concentration of polyphenols (28.3%), followed by total fruit extract (25.5%) and the bark extract (15.7%) (Table 1).The cytotoxic potential of açaí extracts in human tumor cell lines was evaluated by the MTT assay. None of the extracts showed cytotoxicity in the colorectal adenocarcinoma cell lines CACO-2 and HT-29 or in the breast cancer cell line MDA-MB-468 (Figure 1). However, in the MCF-7 cell line, all three extracts reduced the viability of the cells (Figure 2A). Treatment with 20 μg/ml of the bark extract for 48 h significantly reduced MCF-7 viability (p < 0.05) when compared to control cells. A concentration of 40 μg/ml caused a cytotoxic effect at 24 h of incubation (p < 0.05), which increased after 48 h of incubation (p < 0.01).

**Table 1:** Estimation of total polyphenol concentration in juçara hydroalcoholic extracts

***Euterpe oleracea *** **Mart. extract**	**Polyphenol concentration**
Seed	28,3%
Bark	15,7%
Total fruit	25,5%

Treatment with 10 μg/ml for 48 h with seed extract caused significant reduction in MCF-7 viability (p < 0.05). Concentrations of 20 and 40 μg/ml after 24 and 48 h of treatment significantly decreased the viability of MCF-7 cells (p < 0.01). A similar response was observed in MCF-7 cells after treatment with the total fruit extract.To compare the cytotoxic potential among the three extracts, we performed statistical analysis comparing the extracts at the same concentration (Figure 2B). Treatment of cells with the total fruit and seed extracts at a concentration of 10 μg/ml for 24 and 48 h was significantly more effective in reducing the viability of MCF-7 cells than the bark extract (p < 0.001). Twenty-four hours of treatment with 20 μg/ml of the seed or total fruit extract was more effective than treatment with the same concentration of the bark extract (p < 0.01). However, after 48 h, the seed extract was significantly more effective against the MCF-7 cell line than total fruit and bark extracts (p < 0.01). At 40 μg/ml, the extracts were equally effective in reducing the viability of the cells. Since only the MCF-7 cells responded to açaí extracts, all the subsequent assays were performed with MCF-7 cells.

### Açaí extracts caused morphologic alterations in MCF-7 cells

**Figure 3:**
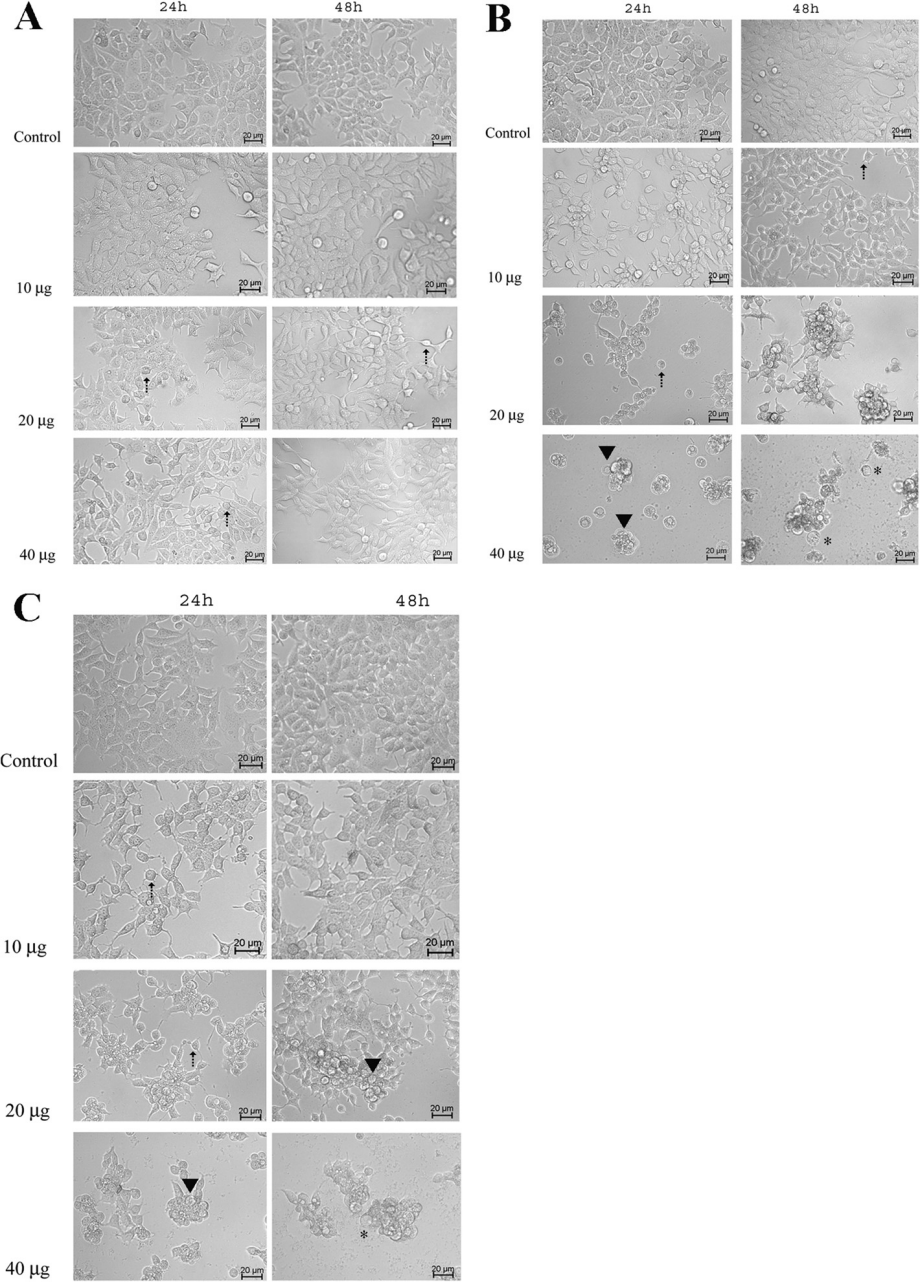
**Morphology analysis by phase-contrast microscopy of MCF-7 cells treated with the bark (A), seed (B), or total fruit (C) extract.** Bark extract treatment caused cell shrinking (arrow). The seed extract caused more dramatic changes in MCF-7 cell morphological features, such as cell shrinking (arrow), membrane blebbing (arrowhead), and cell lysis (asterisk). The same alterations were observed in cells treated with the total fruit extract.

**Figure 4:**
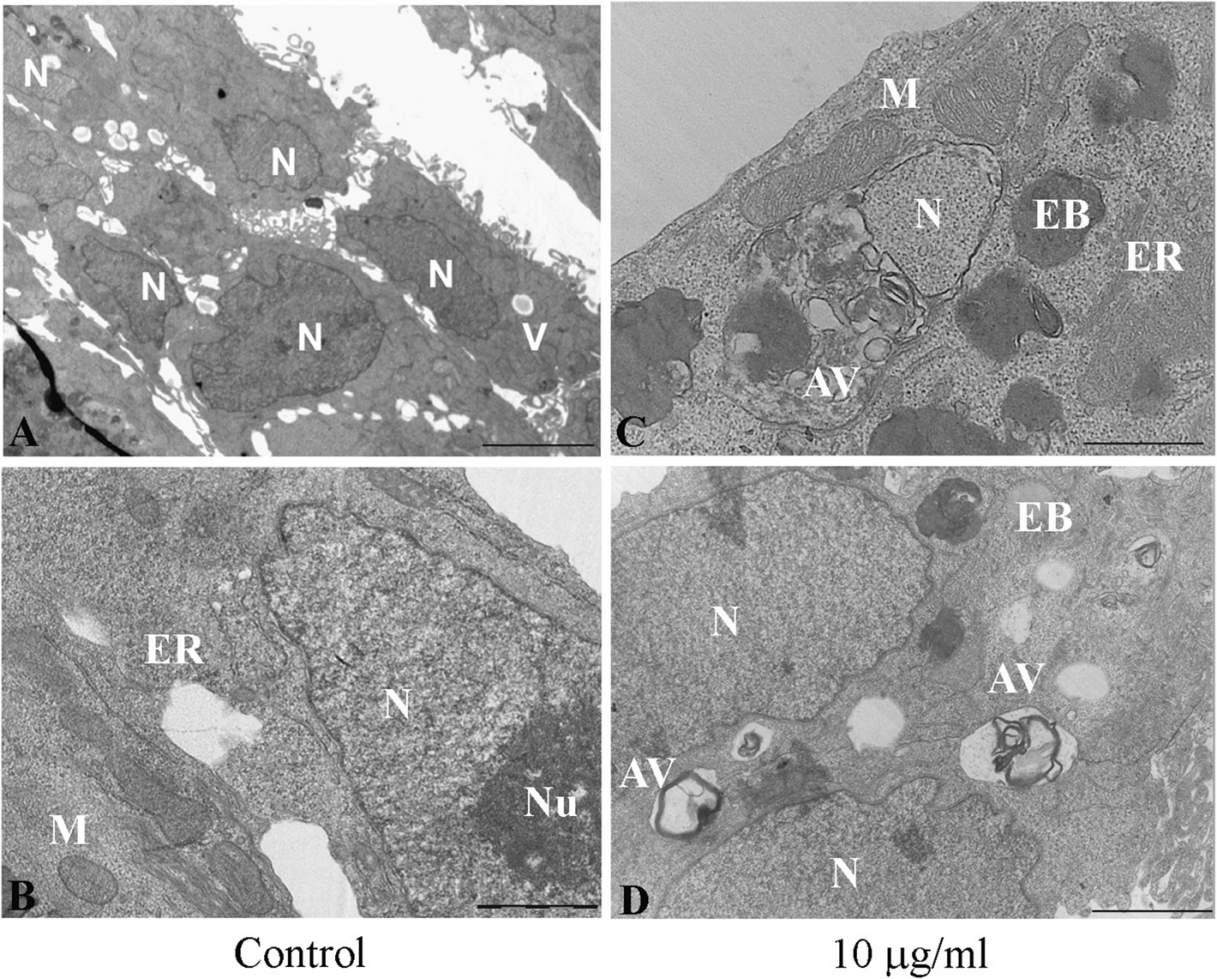
**Morphologic analysis by transmission electron microscopy of control cells and cells treated with 10 μg/ml of seed extract for 24 h. (A)** Representative image of control cells. Some vesicles are present in the cytoplasm. **(B)** Control cells at a higher magnification. **(C)** and **(D)** Treated cells. Autophagic vacuoles and electron-dense bodies can be observed in the cytoplasm. The nucleus in **(D)** is normal. Bar = 1 μm. N = nucleus, Nu = nucleolus, M = mitochondria, V = vesicle, ER = endoplasmic reticulum, AV = autophagic vesicle, EB = electron-dense body.

The effects of açaí extracts on the morphological features of MCF-7 cells were investigated by phase-contrast microscopy and transmission electron microscopy (TEM). Phase-contrast microscopy showed a decrease in cell density in the açaí extract-treated MCF-7 cells, as well as cell rounding and shrinking, membrane blebbing, and cell lysis with apparent loss of cytoplasmic content (Figure 3). The bark extract caused discrete cell rounding and shrinking in MCF-7 cells at concentrations of 20 and 40 μg/ml (Figure 3A). The morphologic alterations caused by seed and total fruit extracts from açaí were more severe than those caused by the bark extract. When used at 10 μg/ml, the seed extract caused a decrease in cell density and cell rounding. Treatment with 20 and 40 μg/ml of seed extracts caused a more accentuated decrease in cell density, cell shrinking, membrane blebbing, and apparent cell lysis (Figure 3B). Similar alterations were observed in MCF-7 cells treated with the total fruit extract (Figure 3C).Detailed morphologic analysis of MCF-7 intracellular organelles was performed by transmission electron microscopy after treatment with 10 μg/mL of seed extract for 24 h. Electron microscopy images of control cells at low magnification showed large nuclei and vesicles in the cytoplasm (Figure 4A). At higher magnification, it was possible to observe intact mitochondria, endoplasmic reticulum, and a double-membrane nuclear membrane (Figure 4B). Seed-extract-treated MCF-7 cells showed the presence of intracellular structures such as autophagic-like vacuoles and electron-dense bodies (Figure 4C and D). The nuclei in seed extract-treated MCF-7 cells were apparently normal, with no evidence of apoptosis (Figure 4D).

### Seed hydroalcoholic extract from açaí caused autophagy in the MCF-7 cell line

**Figure 5:**
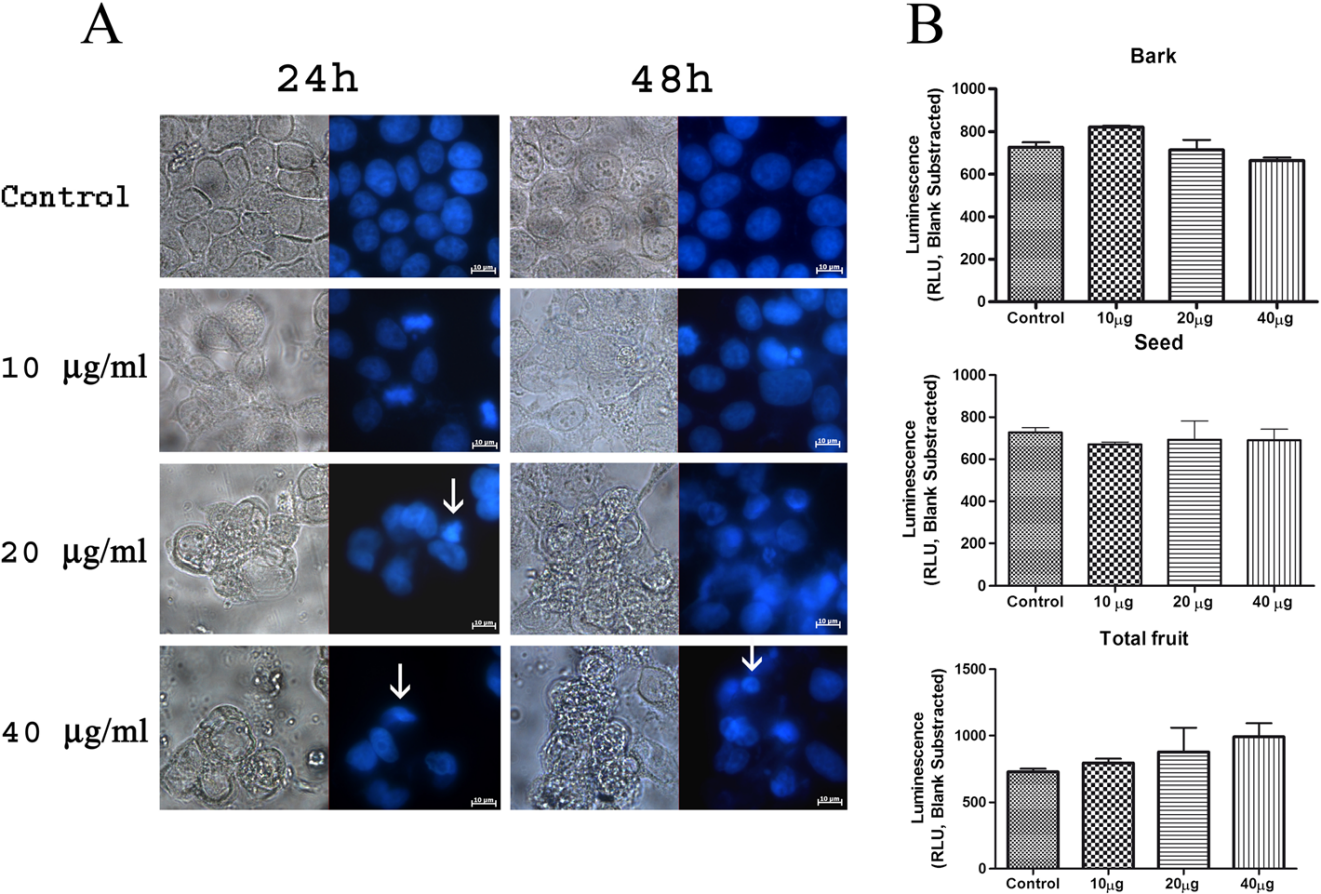
**The seed extract did not caused apoptosis on MCF-7 cells. (A)** Nuclear morphology analysis by DAPI stain. The seed extract caused a few nuclear alterations such as nuclear shrinking (arrow). There was no evidence of apoptosis. **(B)**  Caspase-Glo^®^ 3/7 luminescent demonstrated that none of the extracts increased the activity of caspase 3 and caspase 7 when compared to control cells after 24 h of treatment.

We used a DAPI staining assay and a Caspase-Glo^®^ 3/7 luminescent assay to monitor the events related to cell death via apoptosis after açaí treatments. By using DAPI staining, we observed some nuclear alterations such as volume decrease after seed extract treatment, but we did not detect apoptotic bodies or nuclear fragmentation (Figure 5A). Additionally, the Caspase-Glo™ assays did not show lower caspase 3 and caspase 7 activity in MCF-7 cells treated with the seed, bark, or total fruit extract of açaí, as compared to the activity in control cells (Figure 5B).

**Figure 6:**
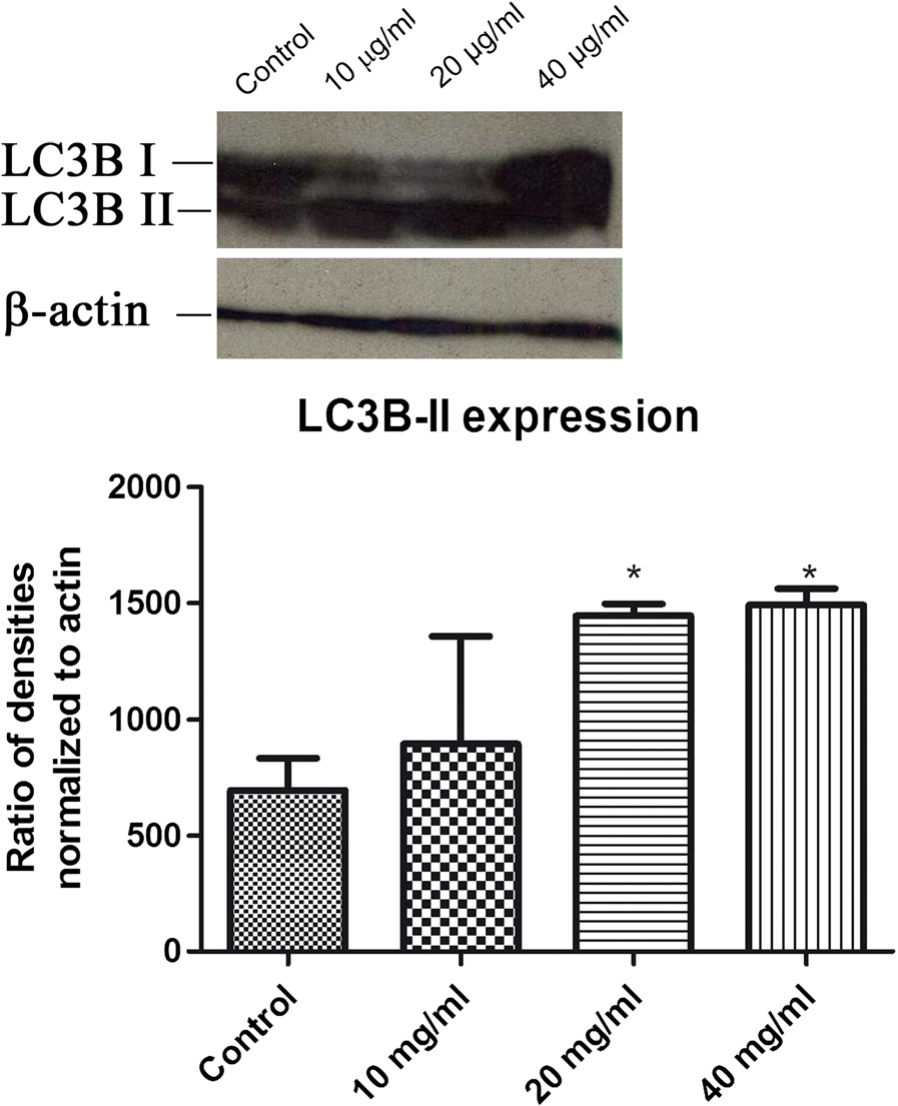
**LC3B-II expression increased in cells treated with 20 and 40 μg/ml seed extract.** *p < 0.05. One-way analysis of variance (ANOVA) followed by Dunnett’s *post hoc* test.

TEM analysis demonstrated the presence of autophagic vacuoles after treatment with the seed extract (Figure 4), and we confirmed these results by western blot analysis performed using anti-LC3B, an antibody that recognizes two forms of LC3B (LC3B-I and LC3-II). Figure 6 shows a significant increase in LC3B-II levels at 24 h after treatment with 20 and 40 μg/ml of seed extract (p < 0.05). LC3B-II protein levels are associated with autophagosome membranes and correlate with the extent of autophagosome formation [13].

## Discussion

In spite of the vast efforts in oncology research and advances in treatment, cancer remains the leading cause of death worldwide [14]. In Brazil, cancer was responsible for more than 178,990 deaths in 2010 [15]. Studies suggest that one-third of cancer deaths could have been avoided by better eating habits and that the diet is related to a variety of human disease conditions such as cancer, hypertension, diabetes, and chronic and acute inflammation [16,17].

Açaí, like other berries and drupes, is rich in polyphenols, anthocyanin, anthocyanidins, and other flavonoids [7,10,18,19], and studies have shown that these substances possess anti-cancer activity in the oral cavity, esophagus, and colon and against leukemia [2,10,11]. However, the cellular mechanisms underlying these activities remain unclear. Since açaí is a frequently consumed plant in the Amazon region, we sought to elucidate the antitumorigenic potential of this drupe in the case of two cancers, colon and breast cancer, with a high prevalence in Brazil.

In the present study, we tested several cell lines and found that the MCF-7 cell line was sensitive to açaí extracts. MCF-7 cells differ from HT-29 and Caco-2 cells with respect to the organ of origin and from MDA-MB-468 by the estrogen receptor status. Estrogen receptor is present in MCF-7 cells and absent in MDA-MB-468 although both are derived from breast cancer [20]. A possible explanation for the specificity for MCF-7 cells is that the cytotoxic effects are caused by substances like lignans present in açaí that may act as phytoestrogens [21]. The phytoestrogens can interact with estrogen receptors and modulate a series of estrogenic and antiestrogenic effects [22,23]. It has been shown that equol, a natural estrogenic metabolite derived from soy isoflavones, enhances tamoxifen’s antitumor activity in MCF-7 breast cancer cells [24]. Whether these substances are responsible for the antitumor activity of açaí remains to be verified since the crude extract has not yet been purified.

We verified that the hydroalcoholic extract from the seed was most potent extract against MCF-7 cells. Analysis of polyphenol content using the Folin-Ciocalteu procedure showed that the seed extract contained the highest concentration of these substances. Studies have described an antitumorigenic effect of polyphenols in several cell lines, such as HeLa, SiHa, and HepG2 cells [25,26]. Therefore, it is likely that the efficacy of the seed extract observed in this study is due to the high concentration of polyphenols. We are currently characterizing the chemical components of the seed extract in order to identify the substance(s) responsible for the antitumorigenic effects.

In order to investigate the type of cell death that occurred in MCF-7 cells, we used DAPI for nuclear visualization and TEM for morphologic analysis. We did not observe nuclear alterations that would be indicative of apoptosis, such as the presence of apoptotic bodies or nuclear chromatin condensation. The results of Caspase-Glo^®^ 3/7 luminescent assays were consistent with these results, in that no differences were seen between the caspase 3 and caspase 7 activity of control and treated cells. However, TEM images showed the presence of electron-dense bodies and autophagic vacuoles in the cytoplasm of MCF-7 cells treated with the seed extract. To confirm autophagy, we performed western blot analysis using an LC3B-II antibody, a well-known marker of autophagosome formation. We observed an increase in LC3B-II expression in cells treated with the seed extract. Other researchers also observed autophagy caused by plant extracts treatments on MCF-7 cells [27,28]. Ait-Mohamed et al. [27] observed that acetonic extract of *Buxus sempervirens* induced autophagy in MCF-7 cells. They observed upregulation of Beclin-1 and downregulation of Survivin and p21. In another study [28] the authors demonstrated that acetone and ethyl acetate extracts from *Eupatorium odoratum* caused autophagic cell death on MCF-7 cell line by a still unknown mechanism. Tsuyuki et al. observed that anthocyanidins caused autophagy but not apoptosis in HeLa cells, a cervical cancer cell line [29]. The leaf water extract of *Solanum nigrum* Linn, rich in polyphenols and anthocyanidins, has been shown to cause autophagy in the AU565 cell lineage [30]. Furthermore, the beneficial effects of resveratrol, a polyphenolic compound found in grapes, have been demonstrated in human subjects and have been attributed to its capacity to promote autophagy [31]. In addition, it was also observed that other phenolic compounds found in red wine were capable of stimulating autophagy [31].

The mechanism whereby cell death of MCF-7 was by autophagy and not apoptosis in our work needs to be determined. Açaí extract may be causing activation of autophagy by a still unknown mechanism. Some speculation based on other works may be done about the possible target of açaí extracts. Gavilán et al. [32] demonstrated that proteasome inhibitors, used for the treatment of some types of cancer, inhibited GSK-3β enzyme regulating autophagy activation in the human breast cancer MCF7 cells. We need to purified the extract and then research the possible target on the cell. It may be an important enzyme like GSK-3β, which is involved in WNT signaling. It is our future goal.

## Conclusion

We conclude that açaí has antitumorigenic potential in the MCF-7 cell line, causing cell viability reduction, morphological alterations, and autophagy induction. Further studies are necessary in order to elucidate the phytochemical(s) responsible for this anticancer activity.

## Competing interests

The authors declare that they have no competing interests.

## Authors’ contributions

MCPC and DS collected the plant and performed the extraction. DFS and FCBV performed the experiments under the supervision of JAMD, MDSBN, and RSM. All the authors analyzed and interpreted the data. FCBV wrote the manuscript draft, which was read and edited by all the authors. All authors read and approved the final version of the manuscript.

## Pre-publication history

The pre-publication history for this paper can be accessed here:

http://www.biomedcentral.com/1472-6882/14/175/prepub
